# Optimizing Surgical Management of Tibial Plateau Fractures: A Comparative Study of Minimally Invasive Versus Open Reduction Techniques

**DOI:** 10.7759/cureus.60078

**Published:** 2024-05-11

**Authors:** Bhaskar Biswas, Ajoy K Halam, Arpita Chowdhury, Tuhin Purkayastha, Santosh Reang

**Affiliations:** 1 Orthopedics, Agartala Government Medical College and Govind Ballabh Pant (GBP) Hospital, Agartala, IND; 2 Orthopedics, Dhalai District Hospital, Dhalai, IND; 3 Anesthesiology, Agartala Government Medical College and Govind Ballabh Pant (GBP) Hospital, Agartala, IND; 4 Orthopedics, ICARE Institute of Medical Sciences and Research and Dr. Bidhan Chandra Roy Hospital, Haldia, Haldia, IND

**Keywords:** postoperative outcomes, orthopedic surgery, fracture fixation, open reduction and internal fixation, minimally invasive surgery, tibial plateau fractures

## Abstract

Background: Tibial plateau fractures pose a significant challenge to orthopedic surgeons due to their complex nature and potential for long-term morbidity. Surgical intervention is often necessary to restore anatomical alignment and optimize functional outcomes. This study aimed to evaluate the efficacy of minimally invasive percutaneous plate osteosynthesis (MIPPO) compared to open reduction and internal fixation (ORIF) in the management of tibial plateau fractures.

Materials and methods: The present hospital-based observational study was conducted at Agartala Government Medical College for two years. Seventy adult patients with tibial plateau fractures were included, with surgical interventions performed based on fracture characteristics. Postoperative outcomes, including knee range of movements, functional recovery, and complication rates, were assessed at six months.

Results: MIPPO demonstrated superior outcomes compared to ORIF, with a higher proportion of participants achieving knee range of movements > 120 degrees (66.7% versus 36%; p = 0.030), excellent functional outcomes (66.7% versus 36%; p = 0.046), and lower postoperative complication rates (2.2% versus 28%; p = 0.001). Fracture union times were significantly shorter in MIPPO (12.49 weeks) when compared to ORIF (14 weeks) (p = 0.009).

Conclusion: MIPPO offers advantages over conventional ORIF in terms of functional recovery and complication rates while demonstrating comparable fracture union times. These findings advocate for the adoption of MIPPO as a preferred surgical technique for tibial plateau fractures.

## Introduction

Tibial plateau fractures are not uncommon, but because of their complexity and potential for severely disabling outcomes if left untreated, they present substantial hurdles in orthopedic practice [1-4]. High-energy trauma, such as falls from great heights or car crashes, is usually the cause of these fractures, which frequently affect the proximal tibia's articular surface [5]. Fractures in this area are especially significant because the tibial plateau, which consists of the proximal end of the tibia, is essential for knee stability and weight-bearing [6].

While treating tibial plateau fractures, surgical care has become the gold standard [7]. Its goals include achieving adequate functional outcomes for the patient and restoring anatomical alignment and stability [8]. The care of tibial plateau fractures involves a complex decision-making process that is frequently impacted by a few variables, such as the type of fracture, the degree of displacement, any accompanying soft tissue damage, the patient's features, and the surgeon's level of experience [9]. A variety of methods, such as open reduction and internal fixation (ORIF), minimally invasive percutaneous plate osteosynthesis (MIPPO), and, in certain situations, external fixation, may be used during surgical intervention [10]. Every technique has pros and cons of its own, and the best technique must be chosen based on the unique needs of each patient and the features of each fracture.

Despite the growing body of literature on tibial plateau fractures, there remains a paucity of high-quality evidence guiding clinical practice, particularly regarding the long-term functional outcomes and complications associated with different surgical techniques. This highlights the need for well-designed studies that can provide valuable insights into the efficacy and safety of various treatment modalities, ultimately informing evidence-based decision-making and optimizing patient care [11,12].

The goal of the current study was to thoroughly evaluate the results of surgical treatment for patients who first presented with fractures to the tibial plateau. The research's goals were to assess the functional result and pinpoint any issues that the study group had after undergoing surgery to treat tibial plateau fractures. The evaluation of functional outcomes will include measures such as range of motion, weight-bearing capacity, and return to pre-injury activities. This will give a thorough picture of the patient's rehabilitation and quality of life following surgery [13-15].

To better understand the safety profile of the surgical treatments under examination, the study also examined the postoperative consequences, such as infection, implant failure, malunion, and nonunion. A crucial stage in the healing process is fracture union, which denotes effective healing and the return of skeletal integrity [16]. Clinical evaluation will include objective measures including pain levels, functional status, and weight-bearing capacity, while radiological assessment will comprise routine follow-up imaging to track the healing of fractures. Through the clarification of the fracture union time course and the identification of variables that impact healing rates, our goal is to enhance patient outcomes and reduce the likelihood of problems by optimizing postoperative care measures and facilitating prompt rehabilitation treatments [17].

This study aims to provide an extensive evaluation of the results of surgical care for patients who have fractures of the tibial plateau, considering important factors including fracture union time, complication rates, and functional recovery. We seek to improve clinical outcomes and the quality of life for those who suffer from tibial plateau fractures by putting light on these crucial characteristics and expanding our knowledge of the best ways to treat them.

## Materials and methods

This non-randomized, prospective study was conducted as a cross-sectional time dimensional study, within the framework of a hospital-based observational investigation. The research was carried out at Agartala Government Medical College, located in Agartala, West Tripura, India. The study was conducted over a period of two years, with one year and six months dedicated to data collection and six months allotted for follow-up assessments.

Ethical approval was obtained from the Institutional Ethics Committee of Agartala Government Medical College with reference number F.4(5-244)/AGMC/Academic/IEC Certificate/2021/7188 before conducting the study, ensuring adherence to ethical guidelines throughout the research process. Informed written consent was taken from each patient before data collection.

The study population comprised all patients with tibial plateau fractures in adults attending the Department of Orthopaedics at Agartala Government Medical College and Govind Ballabh Pant (GBP) Hospital, Agartala, West Tripura, India. Inclusion criteria encompassed patients with all types of closed tibial plateau fractures above 18 years of age of both sexes. Exclusion criteria included skeletally immature individuals, open fractures of the tibial plateau, fractures associated with knee dislocation, and patients with fractures associated with the ipsilateral femur, tibia, and foot.

All cases of tibial plateau fractures satisfying the inclusion criteria and admitted to the Orthopaedics Department of Agartala Government Medical College and GBP Hospital over a period of one year and six months were included in the study. Given the historical data indicating an average of approximately 70 cases enrolled in the inpatient department register over the past three consecutive years, a census sampling technique was employed to ensure the inclusion of all eligible participants.

Study procedure and assessment

The initial workup of patients involved a comprehensive assessment according to the Advanced Trauma Life Support (ATLS) guidelines, including general and systemic examinations, local examination of the injury site, stabilization with intravenous fluids and oxygen, and thorough assessment for associated injuries. A musculoskeletal examination was conducted to rule out associated fractures and injuries, and demographic information such as age, sex, mechanism of injury, and time elapsed between injury and arrival was documented.

After obtaining clearance from the Institutional Ethics Committee and written informed consent from participants, data collection commenced. A total of 70 adult patients with tibial plateau fractures admitted to the orthopedic ward were recruited for the study. Comprehensive history-taking and clinical examinations, including general, systemic, and local evaluations, were performed. The diagnosis was confirmed using plain radiographs, including anteroposterior and lateral views of the knee joint, with non-contrast computed tomography (NCCT) and, in selected cases, with the help of magnetic resonance imaging (MRI). Routine investigations were conducted, including complete blood count, liver and kidney function tests, serum electrolytes, blood sugar levels, coagulation profile, serological tests, chest X-ray, electrocardiogram, and echocardiography in selected cases.

In this non-randomized study, the surgical procedure was selected based on the surgeon's discretion. It is impractical to choose the minimally invasive percutaneous plate osteosynthesis (MIPPO) technique for cases of comminuted fractures having several fragments. Open reduction and internal fixation (ORIF) were performed in simple types of fracture and in certain cases of severely comminuted fractures having multiple fragments where MIPPO was not possible to accomplish accurate reduction. MIPPO was performed in all types of tibial plateau fractures except type VI and a few cases of type IV as per the indication of this special technique. Patients underwent surgical procedures with various forms of implants under epidural anesthesia. Antibiotics were administered preoperatively and continued postoperatively. Preparation of the skin was done with 10% povidone-iodine and spirit, followed by draping with adequate sheets. The surgical approach employed included anterolateral, minimal access anterolateral, and posteromedial approaches, with specific techniques and precautions detailed for each approach. Implants utilized included 3.5 mm T and L buttress plates, pre-contoured proximal lateral tibial plates, 4.5 mm limited contact dynamic compression plate (LC-DCP), lateral tibial head buttress plates, and 6.4 mm cancellous cannulated (CC) screws.

In the anterolateral approach, the patient was placed in a supine position on a radiolucent table with a firm wedge below the flexed knee of approximately 60 degrees. S-shaped incision was made starting approximately 3-5 cm proximal to the joint line, staying just lateral to the border of the patella tendon. The incision was curved anteriorly over Gerdy's tubercle and then extended distally, staying 1 cm lateral to the anterior border of the tibia. The knee joint capsule was incised longitudinally taking care of the lateral meniscus, and the fascia overlying the tibialis anterior muscle was incised carefully. The fracture was reduced, the depressed articular part was elevated under fluoroscopy by bone tamp through a lateral window, and the gap was filled up with autologous or artificial bone graft. The fracture was fixed with a pre-contoured proximal lateral tibial plate or T or L buttress plate. The wound was closed in layers.

In minimally invasive percutaneous plate osteosynthesis (MIPPO), the patient was positioned in the same way as in the anterolateral approach mentioned aforesaid. Two incisions were made: one proximally, starting from proximal and lateral to Gerdy's tubercle, extending distally in a curvilinear way for approximately 5-6 cm, and second distally, 5-6 cm longitudinal incision approximately 2 cm lateral to the tibial crest and parallel with it. Fracture reduction was achieved by indirect reduction techniques with the help of pointed reduction forceps, external fixators, articular tensioning devices, or bone spreaders. A tunnel was made submuscularly with the help of Cobb's elevator. The plate was passed through this tunnel with the help of an external plate holder and fixed with screws on either side under fluoroscopic guidance.

In the posteromedial approach, the patient was placed supine on a radiolucent table, with a sandbag beneath the contralateral hip to roll the patient approximately 200 bringing the posteromedial corner of the tibia forward. A longitudinal 6 cm incision was made overlying the posteromedial border of the proximal tibia, then subcutaneous fat was incised, and the pes anserinus was divided and retracted. Fracture site reduction was achieved. The medial column of the tibia was fixed with T or L buttress plate and 6.4 mm CC screws. Posteromedial fragments may sometimes require fixation with an additional plate (distal radius locking plate). The wound was closed in layers.

**Figure 1:**
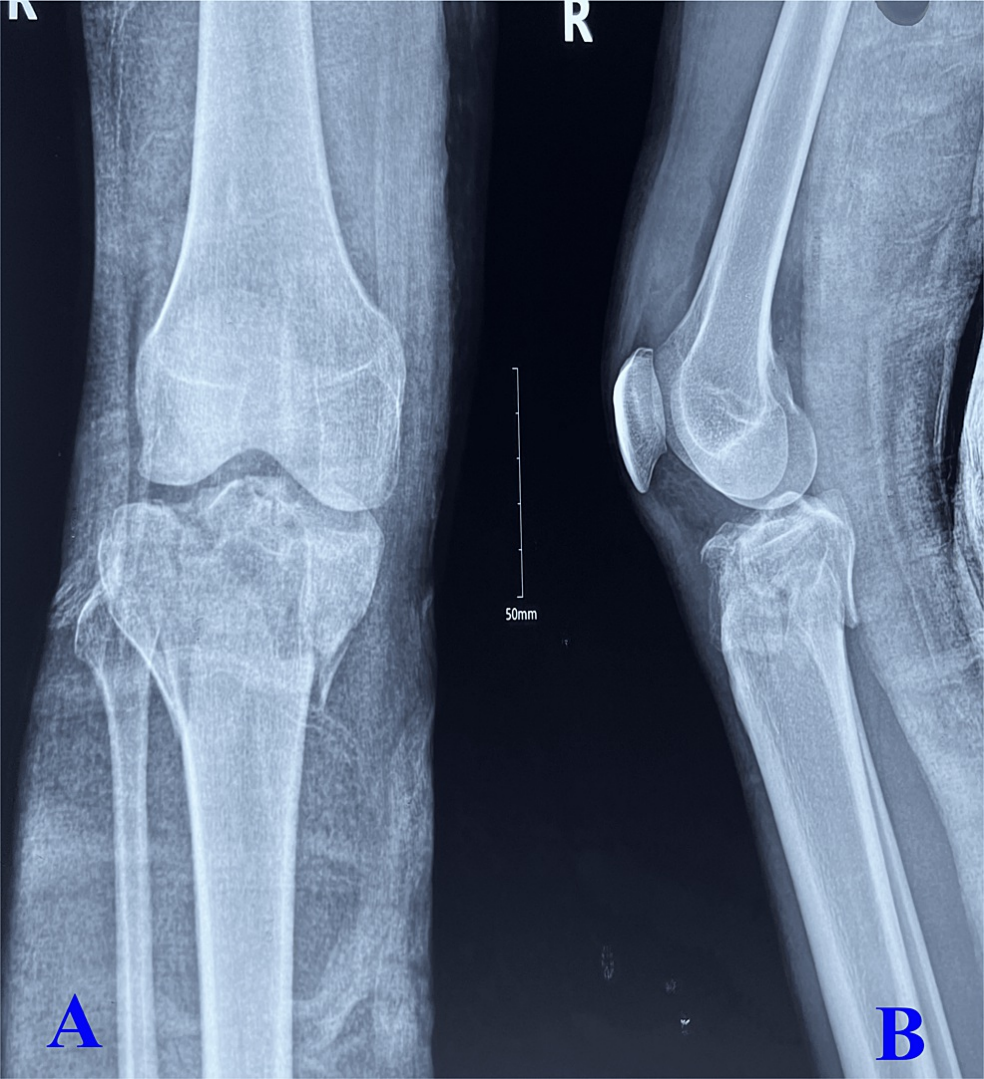
Preoperative X-rays of Schatzker type VI fracture showing the anteroposterior (A) and lateral (B) views A: The anteroposterior view shows a fracture of the medial and lateral column of the proximal tibia with metaphyseal and diaphyseal discontinuity. B: The lateral view shows a fracture of the posterior segment/part of the proximal tibia.

**Figure 2:**
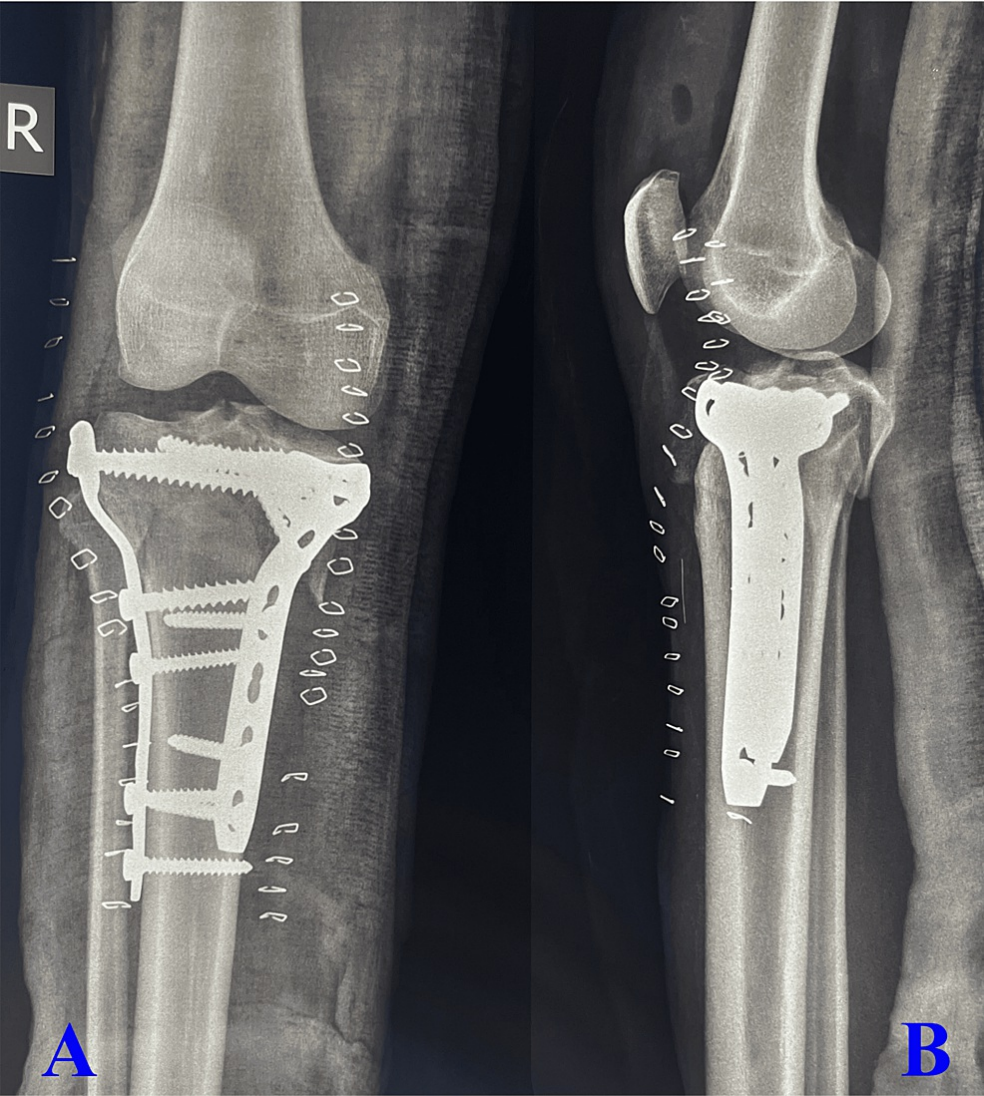
Postoperative X-rays showing the anteroposterior (A) and lateral (B) views A: The anteroposterior view shows adequate reduction of both the column with lateral and medial column plate (3.5 mm pre-contoured proximal lateral tibial plate and 3.5 mm T buttress plate for medial column). B: The lateral view displays adequate reduction and stabilization of the posterior segment of the proximal tibia (T buttress plate).

**Figure 3:**
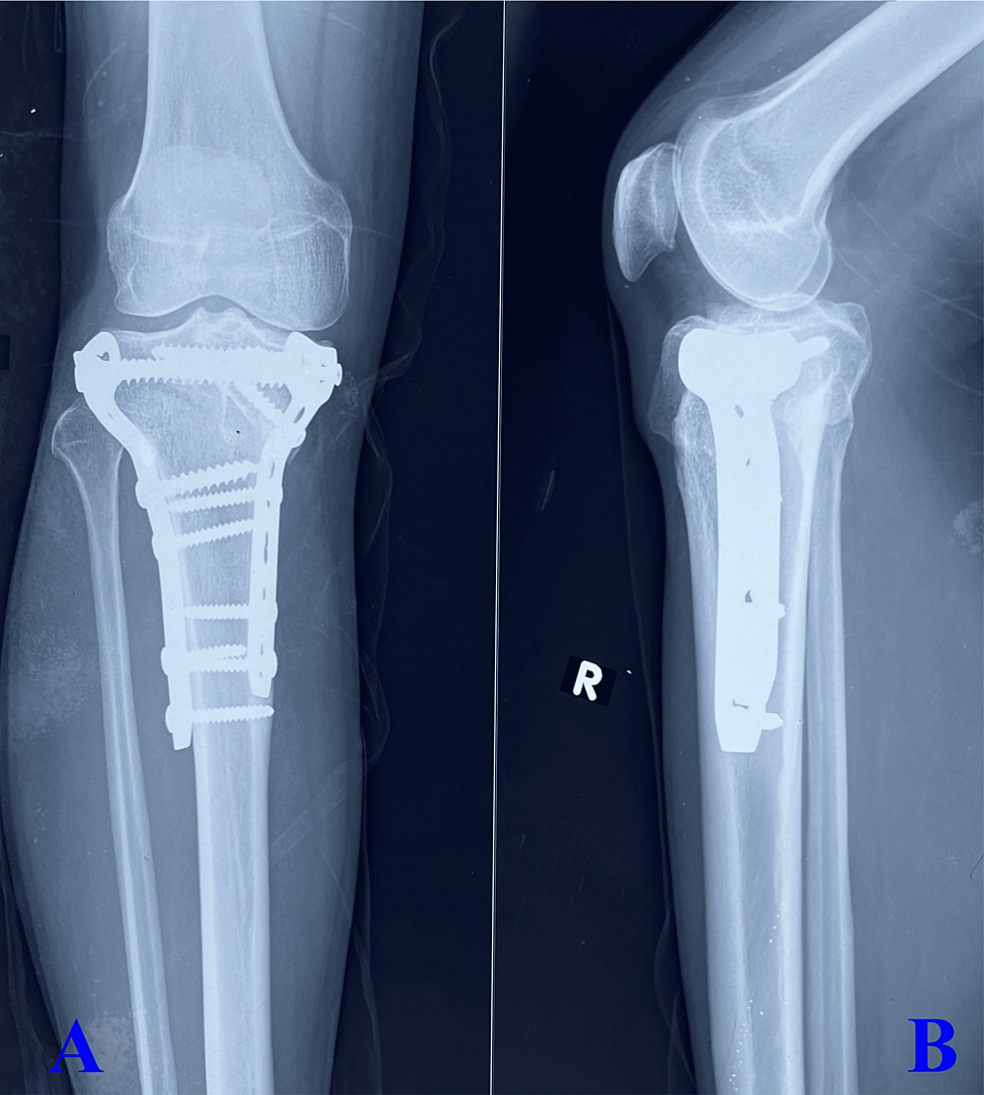
Follow-up X-rays at six months showing the anteroposterior (A) and lateral (B) views A: The anteroposterior view shows fracture union in both medial and lateral columns of the tibia with maintained articular integrity. B: The lateral view shows united posterior segment of the proximal tibia with maintained articular integrity.

Surgical procedures are based on the preoperative images as depicted in Figure 1, in which the anteroposterior view (Figure 1A) shows fracture of the medial and lateral column of the proximal tibia with metaphyseal and diaphyseal discontinuity and the lateral view (Figure 1B) shows fracture of the posterior segment/part of the proximal tibia. Surgical procedures incorporated adequate reduction of both the column with lateral and medial column plate (3.5 mm pre-contoured proximal lateral tibial plate and 3.5 mm T buttress plate for medial column) as depicted in the anteroposterior view of the image (Figure 2A) and adequate reduction and stabilization of the posterior segment of the proximal tibia (T buttress plate) in lateral view (Figure 2B). All patients were followed up after six months of surgical intervention as illustrated in Figure 3, which shows fracture union in both the medial and lateral column of the tibia with maintained articular integrity in the anteroposterior view (Figure 3A) and united posterior segment of the proximal tibia with maintained articular integrity as displayed in lateral view (Figure 3B).

Postoperative pain management was provided, and the affected limbs were immobilized with an above-knee plaster of Paris (AK-POP) slab in selected cases. Active range of motion exercises were initiated early, and patients were instructed in protected weight-bearing techniques. Postoperative follow-up included regular assessments, X-rays, wound inspections, and suture removal as necessary.

Patients were reviewed at the first, second, third, and, finally, sixth months, with X-rays; anteroposterior and lateral views were taken to assess fracture union progression. Functional outcomes were evaluated at the same intervals as stated earlier using the Ranson score, which incorporates subjective patient scores for pain, walking capacity, clinical signs, total range of motion, and stability.

Statistical analysis

Data analysis included proportions, means, standard variations (SD), and other relevant statistical tests using software such as Statistical Package for the Social Sciences (SPSS) version 25 (IBM SPSS Statistics, Armonk, NY). Inferential statistics were done using the Chi-square test in the case of qualitative variables and the Student's unpaired t-test in the case of quantitative variables.

## Results

The data was collected from a total of 70 participants. The mean age of the study participants was 36.43 ± 10.15 years. The median (interquartile range (IQR)) age was found to be 33 (29.75-43) years. The minimum and maximum ages were 21 and 67 years, respectively, with a range of 46 years. The characteristics of the study participants are illustrated in Table 1.

**Table 1:** Characteristics of the study participants

Variables		Number	Percentage (%)
Age group in years	21-30	19	27.1
	31-40	29	41.4
	41-50	14	20.0
	51-60	5	7.2
	61-70	3	4.3
Sex	Males	47	67.1
	Females	23	32.9
Mode of injury	Fall	24	34.3
	Road traffic accident	46	65.7

Most study participants were in the age group of 31-40 years (41.4%), followed by the age group of 21-30 years (27.1%). The distribution across other age groups was as follows: 20% in the 41-50 age group, 7.2% in the 51-60 age group, and 4.3% in the 61-70 age group. Regarding gender distribution, the majority were males, constituting 67.1% of the participants, while females accounted for 32.9%. In terms of the mode of injury, road traffic accidents were the most common, representing 65.7% of cases, followed by falls, which accounted for 34.3% of cases.

Table 2 presents the distribution of fracture types and methods of reduction among the study participants. Schatzker's fracture types were categorized into six types, with type II fractures being the most prevalent (41.4%), followed by type I (27.1%). The least common were type III (1.4%) and type IV (2.9%). In terms of reduction methods, the majority underwent minimally invasive percutaneous plate osteosynthesis (MIPPO), constituting 64.3% of cases, while open reduction and internal fixation (ORIF) were performed in 35.7% of cases. The anterolateral approach was the most frequently employed surgical approach (67.1%), followed by posteromedial (15.7%) and combined approaches (17.1%).

**Table 2:** Fracture types and methods of reduction ORIF: open reduction and internal fixation, MIPPO: minimally invasive percutaneous plate osteosynthesis

Variables		Number	Percentage (%)
Schatzker's fracture types	Type I	19	27.1
	Type II	29	41.4
	Type III	1	1.4
	Type IV	2	2.9
	Type V	11	15.7
	Type VI	8	11.4
Method of reduction and fixation	ORIF	25	35.7
	MIPPO	45	64.3
Approach	Anterolateral	47	67.1
	Posteromedial	11	15.7
	Combined	12	17.1

Table 3 outlines the range of movements, functional outcomes, and postoperative complications observed among the study participants based on the method of reduction. Comparing open reduction and internal fixation (ORIF) to minimally invasive percutaneous plate osteosynthesis (MIPPO), it was found that a higher proportion of participants who underwent MIPPO achieved knee range of movements greater than 120 degrees at six months post-operation (66.7% versus 36%; p = 0.030). Similarly, MIPPO yielded better functional outcomes at six months, with a higher percentage of participants categorized as having excellent outcomes (66.7% versus 36%; p = 0.04). Additionally, postoperative complications such as knee stiffness, wound dehiscence, and delayed union were significantly lower in the MIPPO group compared to the ORIF group (2.2% versus 28%; p = 0.001).

**Table 3:** Range of movements, functional outcome, and postoperative complications among the study participants *Significant P value ORIF: open reduction and internal fixation, MIPPO: minimally invasive percutaneous plate osteosynthesis

Variables		Method of reduction		Chi-square	P value
		ORIF (number (%))	MIPPO (number (%))		
Knee range of movements at 6 months	>120 degrees	9 (36)	30 (66.7)	7.016	0.030*
	90-120 degrees	7 (28)	9 (20)		
	<90 degrees	9 (36)	6 (13.3)		
Functional outcome at 6 months	Excellent	9 (36)	30 (66.7)	6.155	0.046*
	Fair	7 (28)	7 (15.6)		
	Good	9 (36)	8 (17.8)		
Postoperative complications	Absent	18 (72)	44 (97.8)	10.550	0.001*
	Present	7 (28)	1 (2.2)		

Table 4 presents the mean and standard deviation (SD) of fracture union time among the study participants based on the method of reduction. The average time for fracture union was 14 weeks (SD = 2.71) for the open reduction and internal fixation (ORIF) group and 12.49 weeks (SD = 1.96) for the minimally invasive percutaneous plate osteosynthesis (MIPPO) group. The mean fracture union time was significantly shorter in the MIPPO group compared to the ORIF group (t = 2.689, p = 0.009).

**Table 4:** Fracture union among the study participants *Significant P value ORIF: open reduction and internal fixation, MIPPO: minimally invasive percutaneous plate osteosynthesis, SD: standard deviation

Variable		Mean ± SD	t	P value
Fracture union in weeks	ORIF	14 ± 2.71	2.689	0.009*
	MIPPO	12.49 ± 1.96		

## Discussion

Tibial plateau fractures present a significant challenge to orthopedic surgeons due to their complex nature and potential for long-term morbidity if not managed effectively. Surgical intervention plays a pivotal role in restoring anatomical alignment, achieving fracture union, and optimizing functional outcomes [18,19]. In this study, we comprehensively evaluated the outcomes of surgical management in patients with tibial plateau fractures, focusing on functional recovery, postoperative complications, fracture union rates, and comparison of different surgical techniques.

The demographic characteristics of the study population revealed a predominance of males in their fourth decade of life, consistent with previous literature indicating a higher prevalence of tibial plateau fractures among males and middle-aged individuals [20]. Road traffic accidents were identified as the leading cause of injury, highlighting the significant impact of vehicular trauma on musculoskeletal injuries [20,21].

Fracture classification according to Schatzker's system demonstrated a predominance of type II fractures, characterized by lateral split depression of the tibial plateau. This distribution aligns with existing literature, which suggests that type II fractures are the most common subtype of tibial plateau fractures, often resulting from axial loading mechanisms [22-24].

The choice of surgical technique for fracture reduction and fixation remains a topic of debate among orthopedic surgeons. In our study, we observed a higher proportion of patients undergoing minimally invasive percutaneous plate osteosynthesis (MIPPO) compared to open reduction and internal fixation (ORIF). This trend is reflective of the evolving trend toward less invasive surgical approaches, driven by advancements in technology and a desire to minimize surgical trauma and postoperative complications [25,26].

The superior outcomes observed in the MIPPO group, including greater knee range of movements, better functional recovery, lower complication rates, and shorter fracture healing time, suggest that this technique may offer several advantages over conventional ORIF. The minimally invasive nature of MIPPO allows for the preservation of soft tissue integrity and blood supply, reducing the risk of wound complications and promoting early rehabilitation [27]. Additionally, the biomechanical stability provided by locking plates and screws in MIPPO facilitates early mobilization and weight-bearing, contributing to enhanced functional outcomes [28].

However, it is essential to acknowledge the inherent limitations of our study, including its observational nature and relatively small sample size. The retrospective design may introduce selection bias and confounding variables, potentially influencing the interpretation of results. Additionally, the lack of long-term follow-up data limits our ability to assess the durability of surgical outcomes and the incidence of late complications such as osteoarthritis and implant failure.

The comparable fracture union times between the MIPPO and ORIF groups suggest that both techniques are effective in promoting bone healing and achieving fracture union. Despite the slightly longer mean union time observed in the MIPPO group, this difference was not statistically significant, indicating that both approaches offer similar efficacy in facilitating bone consolidation. These findings contradict some previous studies that have suggested faster union times with MIPPO compared to ORIF [29]. However, differences in patient demographics, fracture characteristics, and surgical techniques may account for the variations in reported outcomes.

The choice of surgical approach, including anterolateral, posteromedial, or combined approaches, depends on various factors such as fracture pattern, surgeon preference, and patient-specific considerations [30]. In our study, the anterolateral approach was the most frequently employed, reflecting its versatility and familiarity among orthopedic surgeons. However, it is essential to recognize that each approach has its own set of advantages and limitations, and the selection should be tailored to individual patient anatomy and fracture characteristics.

The strengths of our study include its comprehensive evaluation of surgical outcomes, rigorous data collection methods, and adherence to ethical guidelines. The study highlights the importance of surgical management in achieving satisfactory outcomes in patients with tibial plateau fractures. The limitations of the present study include the limited number of patients in a single institution, relatively short follow-up, exclusion of pediatric patients, open fractures of the tibial plateau, patients with concomitant injuries, and long-term rehabilitation. The findings support the use of MIPPO as a viable alternative to traditional ORIF, offering potential benefits in terms of functional recovery and complication rates. However, further prospective studies with larger sample sizes and long-term follow-up are warranted to validate these findings and refine treatment algorithms for tibial plateau fractures. Additionally, ongoing advancements in surgical techniques and implant designs may continue to shape the landscape of fracture management, ultimately improving outcomes and quality of life for affected individuals.

## Conclusions

The study highlights the significance of surgical intervention in addressing tibial plateau fractures. Minimally invasive percutaneous plate osteosynthesis (MIPPO) emerges as a promising technique, demonstrating superior functional outcomes, lower complication rates, and shorter fracture union times compared to conventional open reduction and internal fixation (ORIF). Further research with larger cohorts and longer-term follow-up is warranted to validate these findings and optimize treatment strategies for tibial plateau fractures.
